# GLP-1 Signalling as a Therapeutic Avenue in Parkinson’s Disease: A Comprehensive Review

**DOI:** 10.3390/ijms262412163

**Published:** 2025-12-18

**Authors:** María Paz Orozco, Valentina Vintimilla Rivadeneira, Jose E. Leon-Rojas

**Affiliations:** 1 NeurALL Research Group, Quito 170157, Ecuador; maria.orozco.gomez@udla.edu.ec; 2 Cerebro, Emoción y Conduca (CEC) Research Group, Escuela de Medicina, Universidad de las Américas (UDLA), Quito 170124, Ecuador; valentina.vintimilla@udla.edu.ec

**Keywords:** Parkinson’s disease, GLP-1, glucagon-like peptide-1 agonists, semaglutide, lixisenatide, exendin-4

## Abstract

Parkinson’s disease (PD) is a complex neurodegenerative disorder characterised by progressive motor and non-motor impairment, in which current therapies remain symptomatic and fail to halt dopaminergic neuron loss. Growing evidence linking metabolic dysfunction, type 2 diabetes, and neurodegeneration has renewed interest in glucagon-like peptide 1 (GLP-1) receptor agonists as potential disease-modifying agents. While several recent reviews have explored the role of incretin-based therapies, the present work provides an integrative perspective by combining a mechanistic analysis of GLP-1 signalling pathways with a model-specific synthesis of preclinical findings and an appraisal of clinical translational relevance. We consolidate evidence across PI3K/Akt, MAPK/ERK, cAMP/PKA–CREB, and AMPK pathways, emphasising their convergence on mitochondrial homeostasis, proteostasis, neuroinflammation, and synaptic resilience. To enhance translational clarity, we summarise preclinical studies across major PD models, evaluate dose comparability and blood–brain barrier penetration, and identify pharmacokinetic and mechanistic factors that may explain divergent clinical outcomes. We also compare the therapeutic potential of key GLP-1 agonists, including exendin-4, liraglutide, semaglutide, lixisenatide, and emerging dual agonists. By integrating biochemical, preclinical, and clinical domains, this review provides a comprehensive framework for interpreting the current evidence and guiding the future development of incretin-based neuroprotective strategies in PD.

## 1. Introduction

Parkinson’s disease (PD) is a progressive neurodegenerative disorder characterized by the loss of midbrain dopaminergic neurons and widespread neuropathological changes, including protein aggregates, primarily composed of α-synuclein [1,2]. These abnormal assemblies undergo oligomerization and misfolding. As a result, they form toxic fibrils that propagate in a prion-like manner across neural circuits, causing harmful effects on the nervous system, commonly associated with neuroinflammation. Furthermore, mitochondrial dysfunction, characterized by an impairment of the electron transport chain and energy deficiency, also contributes to axonal degeneration [1]. Conventional therapies, like levodopa, manage motor symptoms but do not halt neurodegeneration. Despite these treatments’ ability to restore striatal dopamine levels, the pathogenic mechanisms implied in a progressive dopaminergic neuronal loss remain unaltered; current treatments primarily aim to relieve bradykinesia, rigidity, and tremor (i.e., are symptomatic). Nonetheless, long-term use is frequently associated with motor complications, including response fluctuations and dyskinesias [2].

Interestingly, epidemiological and mechanistic links between PD and type 2 diabetes mellitus (T2DM) have suggested that insulin signalling and metabolic dysfunction may contribute to PD pathogenesis [3]. In particular, evidence shows that patients with diabetes treated with incretin-based therapies have a significantly lower incidence of PD [4]. These findings spurred interest in repurposing glucagon-like peptide-1 (GLP-1) receptor agonists, established antidiabetic drugs, as potential neuroprotective agents in PD [5,6]. GLP-1 is an incretin hormone that activates GLP-1 receptors (GLP-1R) to potentiate insulin release, modulating various aspects of cellular metabolism. Notably, GLP-1 receptors are expressed within the central nervous system and can be targeted by GLP-1 analogues capable of crossing the blood-brain barrier [5,7]. A growing body of preclinical evidence demonstrates that GLP-1R agonists (GLP-1RAs) exert broad neuroprotective actions, including support for dopaminergic neuron survival, attenuation of neuroinflammatory processes, enhancement of mitochondrial function, reduction of oxidative stress, and promotion of synaptic plasticity [6,8,9]. For example, GLP-1RAs such as exendin-4 (exenatide) have shown the ability to restore striatal dopamine levels, preserve tyrosine hydroxylase-positive neurons, and alleviate motor deficits in PD animal models [10,11]. Early clinical trials in PD patients provided promising signals of disease modification, with improved motor and cognitive scores that persisted even after drug washout [5,12]. These observations suggest GLP-1 signalling could counteract key pathogenic processes in PD.

Therefore, our review provides a structured overview of the literature on GLP-1 pathway modulation in PD, spanning molecular mechanisms, preclinical findings, and clinical trial outcomes. We emphasize the impact of GLP-1R activation in neuroinflammation, mitochondrial health, dopaminergic neuron survival, oxidative stress, synaptic plasticity, and autophagy. Additionally, we highlight major signalling pathways implicated in these processes, including PI3K/Akt, MAPK/ERK, cAMP/PKA, and AMPK. Furthermore, key GLP-1R agonists under investigation, including exenatide, liraglutide, and semaglutide, are discussed in terms of their pharmacology and evidence of efficacy in PD models and trials.

We conducted a search of the scientific literature across PubMed, Scopus, and Web of Science databases up to August 2025, using combinations of free-text terms and Boolean operators: (“Parkinson’s disease” OR “parkinsonism”) AND (“glucagon-like peptide-1” OR “GLP-1” OR “GLP-1 receptor agonist” OR “incretin” OR “exenatide” OR “liraglutide” OR “lixisenatide” OR “semaglutide” OR “NLY01” OR “PT320” OR “dual receptor agonist”). Reference lists of relevant articles and reviews were also manually screened to identify additional studies. Inclusion criteria were: (1) original research articles in English, (2) experimental or clinical studies evaluating GLP-1 receptor agonists or related incretin-based compounds in Parkinson’s disease or PD models, and (3) studies reporting molecular, behavioural, or clinical outcomes. Exclusion criteria included: (1) studies not involving PD or GLP-1 signalling, (2) commentaries or editorials without primary data, and (3) duplicate or overlapping publications.

This review provides an original and integrative perspective on GLP-1 signaling in Parkinson’s disease by combining three levels of analysis that, to our knowledge, have not been synthesized together in prior publications. First, we consolidate and critically examine the biochemical pathways activated by GLP-1 receptor stimulation, emphasizing their mechanistic convergence on mitochondrial homeostasis, neuroinflammation, proteostasis, and synaptic resilience. Second, we bridge these molecular insights with a structured, model-specific synthesis of preclinical evidence that evaluates not only experimental outcomes but also translational relevance, including pharmacokinetic considerations, blood–brain barrier dynamics, dose comparability across species, and the construct validity of each disease model. Third, we contextualize these findings alongside recent clinical data, highlighting discrepancies between preclinical promise and human outcomes, and identifying patient subgroups and mechanistic domains that may better predict therapeutic responsiveness. Through this combined mechanistic, translational, and clinical framework, the review extends beyond previous summaries of GLP-1 agonists and provides a comprehensive foundation for the rational development and optimization of incretin-based neuroprotective strategies in Parkinson’s disease.

## 2. Functional Mechanisms of GLP-1 Receptors and Agonists in Parkinson Disease

### 2.1. GLP-1 Receptor Distribution in the Brain

GLP-1 receptors (GLP-1Rs) are widely distributed throughout the brain, which underpins the diverse central effects of GLP-1 signalling. GLP-1Rs are expressed in regions relevant to PD, including the striatum and substantia nigra pars compacta (SNpc), specifically in dopaminergic neurons [13,14]. Moderate to low levels of GLP-1R expression have been detected in the hippocampus (CA1 region), cerebral cortex, thalamus, caudate nucleus, globus pallidus, and brainstem nuclei [7,15]. GLP-1Rs are also densely packed in the hypothalamus, specifically, in the supraoptic, paraventricular, and infundibular nuclei [16,17]. Notably, immunohistochemical studies confirm that tyrosine hydroxylase-positive neurons in the SNpc (the cells that degenerate in PD) do express GLP-1Rs [18]. In addition to neurons, GLP-1Rs are predominantly present on glial cells, including astrocytes and microglia [19]. This broad cellular distribution means that GLP-1R agonists could directly modulate neuronal activity as well as glial-mediated neuroinflammatory responses in the brain. Endogenous GLP-1 is produced by brainstem preproglucagon neurons (in the nucleus of the solitary tract) and can act centrally via volume transmission [16,17,18,19].

Peripherally administered GLP-1RAs (e.g., exenatide) have been shown to reach the brain parenchyma. Certain analogues penetrate the blood-brain barrier (BBB) more effectively; for instance, exendin-4 and its analogues display measurable brain uptake, whereas larger analogues like liraglutide and semaglutide cross more slowly or at lower levels in some models [7,20]. Therefore, the blood-to-brain influx rate of exedin-4 remains higher than the other GLP-1 agonists, reflecting differences in their chemical structures [20]. Non-acylated and non-PEGylated GLP-1 agonists, with a negative charge, utilize adsorptive transcytosis to effectively access brain tissue. In contrast, liraglutide’s BBB penetration is limited due to acylation, which promotes oligomer formation, albumin binding, and sequestration within the capillary endothelium, thereby restricting its access and distribution to neural parenchyma [20]. Despite variable BBB permeability, the neuroprotective efficacy of different GLP-1RAs suggests that even those with limited direct brain uptake can exert central effects, possibly via circumventricular organs or indirect mechanisms (e.g., peripheral immune modulation) [7,21]. An example of this is semaglutide, whose elevated albumin affinity impairs BBB penetration. However, this property derives from its long aliphatic chain, enabling it to target GLP-1R-positive brain areas such as the brainstem, septal nucleus, and hypothalamus. Additionally, it affects central nervous system regions through tanycyte passage [22,23].

The presence of GLP-1Rs on vagal afferents and in the area postrema (AP), a circumventricular region located outside the BBB, provides a pathway for peripheral GLP-1 signals to influence the brain. To illustrate, neurons in the nucleus of the solitary tract (NTS) are thought to suppress ingestive behaviour through central GLP-1 production, triggered by vagal sensory information and gustatory input [14]. Furthermore, GLP-1 has an excitatory effect in AP neurons, also implied in food intake regulation [24,25].

Overall, the CNS distribution of GLP-1Rs provides a neuroanatomical basis for targeting this system in PD, with the potential to impact multiple brain regions involved in motor, cognitive, and autonomic aspects of the disease.

### 2.2. Neuroinflammation and GLP-1 Signaling

Neuroinflammation plays a pivotal role in PD progression, characterized by significant microglial activation, which leads to elevated levels of pro-inflammatory cytokines and reactive oxygen species in the nigrostriatal system. GLP-1 receptor agonists have demonstrated robust anti-inflammatory effects in the brain [26,27]. One key mechanism is the modulation of microglial activation states. GLP-1R activation suppresses the production of pro-inflammatory cytokines such as tumour necrosis factor-α (TNF-α), interleukin-1β, and IL-6 by microglia [19]. This results in a shift from a neurotoxic inflammatory state to a more neuroprotective environment. Certainly, in preclinical models, GLP-1RAs prevented the transformation of astrocytes into the A1 neurotoxic phenotype that is induced by activated microglia [28]. For example, the GLP-1RA known as NLY01 (a pegylated exendin-4 derivative) was shown to directly block microglia-mediated conversion of astrocytes into A1 reactive astrocytes, thereby averting the release of neurotoxic factors and protecting neurons [29,30]. On top of that, in an α-synuclein preformed fibril model of sporadic PD, NLY01 not only inhibited astrocytes transformation but also preserved dopaminergic neurons and ameliorated associated behavioural deficits [28].

At the molecular level, GLP-1R stimulation can interfere with inflammatory signalling pathways. Elevated cAMP (from GLP-1R’s G_s coupling) tends to inhibit NF-κB activation in immune cells, reducing the transcription of inflammatory genes [31]. GLP-1RAs have also been reported to inhibit Toll-like receptor (TLR)-induced inflammatory responses in the CNS [32], suggesting a broad dampening of innate immune activation. GLP-1R activation has demonstrated the ability to shift cytokine signalling toward an anti-inflammatory profile. This phenomenon was observed in a recent 2024 preclinical study, in which administration of GLP-1RAs led to a 38 ± 4.2% increase in plasma IL-10 levels. Concurrently, a significant reduction in pro-inflammatory cytokines, such as TNF-α and IL-6, was reported, showing a 42 ± 3.5% decrease compared to baseline values [32,33]. In parallel, upregulation of neurotrophic factors also contributes to inflammation resolution. Exedin-4 exemplifies this effect by promoting the expression of brain-derived neurotrophic factor (BDNF), a protein that facilitates synaptic regeneration and neuronal survival [34,35]. Furthermore, in PD animal studies, these anti-inflammatory effects correlate with reduced nigral neuron loss [3]. Liraglutide treatment in MPTP-lesioned mice, for instance, significantly attenuated microglial activation in the substantia nigra and lowered levels of inflammatory markers, accompanying improvements in motor function [36,37,38]. This finding highlights how GLP-1 signalling through multi-faceted immunomodulation counteracts the chronic neuroinflammation that contributes to dopaminergic neuron injury in PD.

### 2.3. Mitochondrial Dysfunction

Mitochondrial impairment is a central feature of PD pathology, contributing to energy failure and DA neuron death. GLP-1 receptor activation can improve mitochondrial function and biogenesis in stressed neurons. A prominent pathway involved is the PI3K/Akt signalling cascade, which GLP-1RAs trigger in neurons (often via insulin/IGF-1 signalling crosstalk). Activation of PI3K/Akt leads to downstream effects like enhanced activity of the master regulator of mitochondrial biogenesis, PGC-1α (peroxisome proliferator-activated receptor-γ coactivator 1α) [6]. Following GLP-1R activation, AKT is upregulated, resulting in reduced synthesis of pro-apoptotic proteins, such as BiM and FAS. Moreover, it has also been implicated in promoting cell survival by stimulating anti-apoptotic signalling pathways, primarily through elevated cAMP levels [8,39]. Studies show that GLP-1 treatment elevates levels of proteins involved in oxidative phosphorylation and mitochondrial dynamics (e.g., COX 5A, PGC-1α) [9,40]. In PD models, exendin-4 and dual incretin agonists were found to upregulate mitochondrial transcription factors such as NRF1 and SIRT1, promoting mitochondrial biogenesis and enhancing respiratory function [41].

GLP-1RAs also protect neurons from toxin-induced mitochondrial stress. In cellular models, exendin-4 pre-treatment protected dopaminergic cells from rotenone (a mitochondrial complex I inhibitor) by preserving mitochondrial membrane potential and preventing excessive mitochondrial fragmentation [9,42,43]. This was accompanied by an increase in markers of autophagy that clear damaged mitochondria (mitophagy), suggesting GLP-1R stimulation helps remove dysfunctional mitochondria and maintain healthy populations [44]. In vivo, GLP-1 analogues have been shown to restore levels of mitochondrial enzymes in PD models. For example, in MPTP-treated mice, drugs like liraglutide normalized complex I activity and ATP levels in nigral neurons [38].

A 2024 study reported that another GLP-1RA, PT320, delays disease progression in a MitoPark mouse model of PD. PT320 demonstrated promising therapeutic potential, exerting neuroprotective effects by preserving mitochondrial function and ultrastructure [45,46]. Notably, early administration of PT320 preserved mitochondrial integrity by regulating the expression of Opa1, which prevents cytochrome c release via cristae remodelling, and Fis1, a key mediator of mitochondrial fission. These molecular effects maintained mitochondrial homeostasis. Other studies have evidenced its protective role by delaying dopaminergic degeneration, improving striatal dopamine release and reuptake, and enhancing spontaneous motor behaviour in a mouse model of early PD [45,46]. MPTP and oxidative stress exposure induce mitochondrial dysfunction, including morphology changes, such as fragmentation, which result in aberrant ultrastructure and apoptosis via caspase activation. This effect is reversed by the administration of incretin agonists, leading to a more uniform and reduced mitochondrial size [9]. These effects collectively suggest that GLP-1 signalling boosts the energetic capacity of neurons and fortifies them against the mitochondrial toxins and oxidative insults that drive PD neurodegeneration.

### 2.4. Oxidative Stress

Oxidative stress, arising from excess reactive oxygen species (ROS) and impaired antioxidant defences, contributes to the degeneration of dopaminergic neurons in PD. GLP-1R agonists have demonstrated potent antioxidative effects in the CNS [3,5,6]. One mechanism is through upregulating endogenous antioxidant enzymes. GLP-1 signalling activates pathways (such as cAMP/PKA and Nrf2) that boost the expression of antioxidant proteins like superoxide dismutase (SOD), catalase, and glutathione peroxidase [47]. In PD model systems, treatment with GLP-1 analogues reduced markers of oxidative damage. For instance, mice receiving semaglutide or liraglutide after chronic MPTP exposure showed significantly lower levels of lipid peroxidation (malondialdehyde) and protein carbonylation in the brain compared to untreated PD controls [36,44,48]. This indicates mitigation of ROS-induced cellular injury.

Another way GLP-1R activation combats oxidative stress is by improving mitochondrial efficiency (as discussed above), thereby reducing the overproduction of ROS from dysfunctional mitochondria. GLP-1RAs also indirectly reduce oxidative stress by attenuating neuroinflammation; activated immune cells produce ROS and nitric oxide, so dampening microglial activation via GLP-1 can decrease this source of oxidative molecules [40,46,49].

Exendin-4 has been shown to reduce oxidative stress, suppress inflammatory mediators, and modulate the activation of MAPK family kinases, including JNK and p38 MAPK [50]. In addition, it increases glutathione and magnesium superoxide dismutase levels and decreases the expression of pro-apoptotic marker, BAX [42,51]. Combined, these actions protect neurons from oxidative damage. Furthermore, in a mouse model of cerebral ischemia-reperfusion, administration of exendin-4 reduced oxidative DNA damage in brain cells, as indicated by decreased 8-hydroxy-2-deoxyguanosine (a biomarker of oxidative DNA damage) and HHE levels (a major product of lipid peroxidation) [52,53,54]. By bolstering antioxidant responses and limiting ROS generation, GLP-1RAs create a more redox-stable environment for vulnerable neurons in PD.

### 2.5. Dopaminergic Neuron Survival and Neuroprotection

Protecting the survival of dopaminergic neurons in the substantia nigra is a primary goal for disease-modifying PD therapy. GLP-1 receptor stimulation has repeatedly been shown to enhance dopaminergic neuron survival in both toxin and genetic models of PD, partly by maintaining spontaneous firing activity and supporting neuronal function [55]. In a preclinical experimental study, 6 out of 12 neurons were excited by exendin-4, showing a marked rise in firing rate from 2.48 ± 0.29 Hz to 3.61 ± 0.45 Hz [18]. Conversely, when the same neuronal population was exposed simultaneously to exendin, a GLP-1R antagonist, and exendin-4, no significant changes in firing activity were observed. These findings highlight the ability of GLP-1 to increase neuronal excitability in the substantia nigra, contributing to the production of its possible anti-Parkinsonian effects [18]. Furthermore, in rodent studies, systemic administration of GLP-1RAs (exendin-4, liraglutide, etc.) preserved the number of tyrosine hydroxylase (TH)-positive neurons in the SNpc following neurotoxic insults [36]. For example, in a classic 6-hydroxydopamine (6-OHDA) lesioned rat model, exendin-4 treatment attenuated the loss of nigral dopaminergic neurons and maintained striatal dopamine levels, correlating with improved motor function [56]. Similarly, in studies with MPTP-treated mice, once-weekly semaglutide produced greater restoration of nigral TH levels and protection of dopaminergic cell bodies than once-daily liraglutide, demonstrating superior efficacy [5,23]. These interventions also normalized dopamine turnover and receptor expression in the basal ganglia, suggesting preservation of functional dopaminergic transmission.

Mechanistically, GLP-1R activation engages intracellular survival pathways that counteract pro-death signals in neurons. A major pathway is the PI3K/Akt axis, which promotes cell survival; GLP-1RAs strongly activate Akt in dopaminergic neurons, leading to phosphorylation and inactivation of pro-apoptotic factors like glycogen synthase kinase-3β and the Bcl-2-associated death promoter (BAD) [41]. Akt activation also upregulates the expression of anti-apoptotic Bcl-2 family proteins, creating a cell survival bias [57]. Certainly, in models of PD, GLP-1RAs reduced neuronal apoptosis, as evidenced by decreased caspase-3 activation and fewer TUNEL-positive cells in the substantia nigra [13,58]. Another protective mechanism is via neurotrophic support; GLP-1R signalling can induce brain-derived neurotrophic factor (BDNF) and glial cell line-derived neurotrophic factor (GDNF) expression in the brain [59]. Notably, semaglutide increases GDNF levels in the SN and striatum of MPTP mice, which likely contributed to the rescue of dopaminergic neurons [44]. Enhanced trophic support promotes neuronal resilience under stress and may facilitate endogenous repair mechanisms.

Emerging evidence indicates that GLP-1RAs can also stimulate neurogenesis and may support the recruitment of new dopaminergic neurons. Some studies have reported that GLP-1 treatment increases the proliferation of neural progenitors and directs their differentiation towards a dopaminergic lineage [34]. Consistently, a preclinical, in vitro and in vivo experimental study, utilizing SOD1-G93A transgenic ALS mouse model and motor neuron-like cells (NCS-19), respectively, has shown that exendin-4 treatment results in reduced oxidative intracellular stress markers, improved cell survival, and preserved mitochondrial activity, suggesting metabolic stabilization [60]. Likewise, its histological analysis demonstrated that this GLP-1 agonist maintained phenotypic identity and cholinergic function of surviving neurons; preservation of ChAT levels correlates with better neuronal integrity, reduced apoptosis, and decreased glial activity [60].

Conversely, in another preclinical study, also using exendin-4 as a therapeutic alternative for PD, employing an MPTP-induced Parkinson’s disease mouse model found that systemic exendin-4 robustly protected striatal dopaminergic terminals, suppressed microglial activations, preventing inflammatory morphological changes, and inhibited both cytokines at the protein and mRNA level [61].

Therefore, exendin-4 has been shown to protect dopaminergic and cholinergic neurons through overlapping mechanisms involving cAMP/PKA, PI3K/Akt, MAPK/ERK, anti-inflammatory signaling, and cellular protection, benefits that could potentially make this treatment an option for becoming a multi-target disease-modifying treatment in neurodegeneration.

Additionally, in a primary rodent neural model, GLP-1R intervention notably enhanced the proliferative potential of murine neural stem cells, suggesting that the PI3K/AKT signalling pathway promotes neuronal differentiation via the regulation of protein structural integrity and the induction of transcription of the proneural factor Achaete-scute homolog 1 (Ascl1) [41]. Ascl1 promotes neuronal differentiation of neural progenitor cells by activating target genes and driving cell cycle exit. In addition, experimental studies have shown that Ascl1 acts as a dominant factor in the transdifferentiation of non-neuronal cells, such as fibroblasts, into neurons [62].

Through these converging mechanisms (anti-apoptotic signalling, metabolic support, anti-inflammatory effects, neurogenesis promotion, and neurotrophic factor induction), GLP-1R agonists provide several pro-survival benefits for dopaminergic neurons. The net result in animal models is reduced dopaminergic cell loss and sustained motor function. These multifaceted neuroprotective effects underpin the potential of GLP-1-based therapies to slow or even halt the progression of PD in patients, particularly if administered early in the neurodegenerative process.

### 2.6. Synaptic Plasticity and Cognitive Function

Beyond preserving neurons, GLP-1 signalling may improve synaptic plasticity and function, which is relevant to both motor and cognitive symptoms of PD. GLP-1Rs in the brain are known to modulate synaptic transmission and memory processes. The upregulation of neurotrophic tyrosine kinase receptor type 2 and mTOR genes, both of which play key roles in managing synaptic plasticity, has been identified after administering lixisenatide for 40 days in mice with cognitive impairment [10]. In PD contexts, GLP-1R agonists have been found to protect synapses from degeneration and to promote the formation of new synaptic connections (synaptogenesis) [63]. One underlying mechanism involves the cAMP/PKA pathway; activation of GLP-1Rs increases intracellular cAMP, which in turn activates protein kinase A (PKA). PKA can phosphorylate glutamate AMPA receptor subunits (e.g., GluR1 at Ser^845^), facilitating their insertion into synapses and thereby strengthening synaptic currents and long-term potentiation (LTP) [13,18,63]. Indeed, studies in Alzheimer’s models (which are translatable to PD cognitive impairment) showed that GLP-1 analogues prevented oligomeric Aβ-induced loss of synaptic proteins in a PKA-dependent manner, effectively preserving LTP [13,18]. In PD models, exendin-4 has similarly been reported to restore synaptic marker proteins that were reduced by α-synuclein pathology or oxidative stress, suggesting stabilization of synaptic integrity [64].

GLP-1R activation also directly supports neurite outgrowth and synapse formation. In neuronal cultures (PC12 and SH-SY5Y cells), GLP-1 analogues induced robust neurite branching and extension, an effect that requires cytoskeletal reorganization via actin/tubulin polymerization [65,66]. This pro-neuritogenic effect implies that GLP-1 signalling can help re-establish circuit connections or compensate for lost innervation in the brain. Furthermore, by reducing neuroinflammation and oxidative damage, GLP-1RAs create conditions where synapses are less likely to be dismantled [13,67].

Behaviourally, these synaptic benefits translate into improved cognitive outcomes in experimental models. PD patients often have cognitive deficits, and interestingly, the initial open-label exenatide trial reported improved performance on the Mattis Dementia Rating Scale in treated patients compared with controls [5,12]. While that was a small sample, it aligns with preclinical findings that GLP-1R stimulation enhances hippocampal synaptic plasticity and memory [68]. For example, GLP-1R knockout mice have shown impaired LTP and memory formation, whereas GLP-1RAs enhance memory in toxin-induced parkinsonian mice (possibly via effects on the hippocampus and cortex). Overall, GLP-1RAs appear to not only safeguard existing synapses from PD-related insults but also promote adaptive plasticity, which could ameliorate both motor learning and cognitive function [68,69].

### 2.7. Autophagy and Protein Clearance

Impaired proteostasis, including the accumulation of misfolded α-synuclein, is a hallmark of PD. Autophagy is the cell’s waste clearance system that helps remove protein aggregates and damaged organelles [70]. GLP-1 signalling has been implicated in modulating autophagy in beneficial ways for PD. An in vitro experimental study demonstrated that semaglutide exerts neuroprotective effects against 6-OHDA-induced toxicity in human SH-SY5Y neuronal cells by enhancing autophagy, as evidenced by elevated levels of key autophagy markers such as LC3-II/LC3-1, beclin1 and Atg7, along with reduced p62 concentration, a protein that accumulates when autophagy is impaired [48]. GLP-1R activation can enhance autophagic flux to clear toxic proteins while preventing excessive or dysregulated autophagy that might harm cells [9,71]. In a neuronal model of PD, exendin-4 improved autophagy markers and cell survival under rotenone-induced stress, suggesting that GLP-1R stimulation facilitates the clearance of dysfunctional mitochondria and protein aggregates caused by the toxin [43]. Concurrently, by boosting cellular energetic status and mTOR signalling, GLP-1 may avert the maladaptive overactivation of autophagy that can lead to cell death. Indeed, a study reported that a GLP-1 mimetic prevented an “aberrant enhancement of autophagy/mitophagy” in neurons under oxidative stress, thereby protecting axons from degeneration [71]. In vivo evidence reinforces the potential of GLP-1RAs to promote the removal of pathological protein accumulations; in particular, semaglutide-treated MPTP mice have shown reduced α-synuclein accumulation in the brain compared to untreated parkinsonian mice [44]. Similarly, liraglutide decreased the aggregation of toxic proteins in these models [9,36].

These outcomes suggest that GLP-1R activation enhances the efficiency of autophagic clearance of α-synuclein and potentially other cargo, such as dysfunctional mitochondria, in that way reducing the burden of proteinopathy in PD [9,36]. The latter is illustrated in a study that measured the mitochondrial inner membrane (MIM) fusion protein OPA1, which was initially reduced in the MTPT group but was upregulated following GLP-1RA administration. In contrast, the outer membrane (MOM) fusion proteins MFN1 and MFN2 were elevated in the exposed group and decreased after treatment. Although all three proteins contribute to mitochondrial fusion, their differential regulation suggests that complex I inhibition induces an imbalance in the fusion-triggered process, which is partially restored by the GLP-1RA intervention [9,46]. GLP-1RAs might exert these effects via AMPK activation (a known inducer of autophagy) as well as through transcriptional upregulation of autophagy-related genes [72,73]. By fine-tuning the autophagy process, GLP-1RAs can contribute to proteostasis maintenance, which reduces stress on the endoplasmic reticulum and proteasome systems in dopaminergic neurons, ultimately contributing to their survival [74]. Autophagy is a double-edged sword in PD, but GLP-1 seems to tilt it toward neuroprotection rather than neurodegeneration.

### 2.8. L-DOPA-Induced Dyskinesia (LID)

L-3,4-dihydroxyphenylalanine (L-DOPA) is the precursor of dopamine and is the most used medication for PD, at the moment [45]. Nevertheless, chronic administration of L-DOPA in PD patients is habitually associated with the presence of involuntary movements (AIMS), also called levodopa-induced dyskinesia. Certainly, early clinical studies showed that about 20–50% of L-DOPA-medicated patients experience dyskinesia within 5 years after initiating its treatment; the severity of the symptoms correlates with the length of its usage [75,76,77]. DA agonists can overstimulate dopamine receptors in both the direct and indirect striatopallidal pathways in the lesioned brain. Consequently, this is thought to lead to reduced neuronal firing in the subthalamic nucleus and internal globus pallidus, resulting in increased involuntary movements [75,76,77].

6-OHDA rat models are widely used to examine AIMs. The injection of 6-hydroxydopamine causes the loss of dopaminergic neurons, resulting in unilateral DA depletion in the striatum. In order to assess dyskinesia, rats are observed over a period at regular intervals following L-DOPA administration [75,76,77]. Three subtypes of AIMs are typically determined: random uncontrollable movement of the forelimb contralateral to the lesion, excessive jaw movements, and dystonic postures or choreiform twisting of the neck and upper body [75,76,77]. The ALO score represents the cumulative dyskinesia severity, typically from 1 (present for less than 30 s) to 4 (present throughout a minute and not suppressible). Favorably, these types of experimental studies have been crucial for testing neuroprotective agents. For instance, 3-week co-administration of PT320, a sustained-release formulation of exendin-4, was shown to notably decrease the AIM scores for ALO (*p* = 0.031), limb (*p* = 0.036), and orolingual (*p* = 0.008) movements [45]. This study represents the general neuroprotective mechanisms of GLP-1RAS, which involve activating the PI3K/Akt pathway, so it modulates PD disruption by enhancing synapse formation and autophagy while inhibiting the accumulation of alpha-synuclein [45]. These mechanisms are relevant since LID may involve disrupted signaling with the basal ganglia circuits.

## 3. GLP-1 Neuroprotective Signalling Pathways

**Figure 1:**
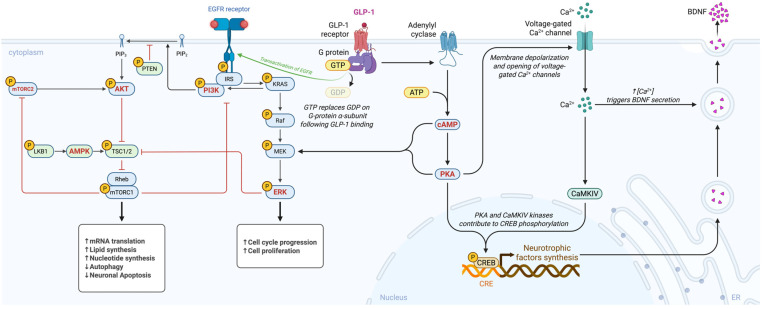
Mechanistic overview of GLP-1 receptor signalling in Parkinson’s disease. AMP-activated protein kinase (AMPK), adenosine triphosphate (ATP), brain derived neurotrophic factor (BDNF), calcium ion (Ca2plus), calcium calmodulin dependent protein kinase IV (CaMKIV), cyclic adenosine monophosphate (cAMP), cAMP response element (CRE), cAMP response element binding protein (CREB), epidermal growth factor receptor (EGFR), extracellular signal regulated kinase (ERK), guanosine diphosphate (GDP), glucagon like peptide 1 (GLP minus 1), guanosine triphosphate (GTP), insulin receptor substrate (IRS), Kirsten rat sarcoma viral oncogene homolog (KRAS), liver kinase B1 or serine threonine kinase 11 (LKB1), mitogen activated protein kinase kinase (MEK), mechanistic target of rapamycin complex 1 (mTORC1), mechanistic target of rapamycin complex 2 (mTORC2), phosphatidylinositol 4,5 bisphosphate (PIP2), phosphatidylinositol 3,4,5 trisphosphate (PIP3), phosphoinositide 3 kinase (PI3K), protein kinase A (PKA), phosphatase and tensin homolog (PTEN), rapidly accelerated fibrosarcoma kinase (Raf), Ras homolog enriched in brain (Rheb), and tuberous sclerosis complex 1 and 2 (TSC1/2). Created in BioRender. Américas, U. (2025) https://BioRender.com/3jfxi8g.

GLP-1 receptor activation engages multiple intracellular signalling cascades that converge on neuroprotection. Key pathways and their roles will be discussed below and in Figure 1.

**PI3K/Akt Pathway**: GLP-1R stimulation triggers phosphoinositide 3-kinase (PI3K), leading to activation of Akt (protein kinase B). Akt promotes cell survival by inhibiting pro-apoptotic proteins (e.g., Bax, BAD) and upregulating anti-apoptotic factors such as Bcl-2 [39,78]. In addition, Akt enhances mitochondrial health by stimulating biogenesis and efficiency through regulation of downstream targets, including PGC-1α and NRF-1, and by inhibiting GSK-3β activity [79,80,81]. In PD models, the PI3K/Akt pathway has been strongly implicated in mediating exendin-4’s anti-apoptotic and neurorestorative effects [6,82].

**MAPK/ERK Pathway**: The extracellular signal-regulated kinase (ERK) branch of the mitogen-activated protein kinase (MAPK) cascade is activated downstream of GLP-1R through cAMP or β-arrestin signalling [13,83]. ERK activation supports neuronal survival and synaptic plasticity. GLP-1RAs stimulating ERK have been shown to increase CREB (cAMP response element-binding protein) activity and expression of neurotrophic factors like BDNF [84,85] This pathway likely contributes to synaptic repair, neuritogenesis, and cognitive improvements observed with GLP-1 analogues [86].

**cAMP/PKA Pathway**: As a Gs-coupled receptor, GLP-1R robustly elevates intracellular cyclic AMP (cAMP); cAMP directly activates protein kinase A (PKA) as well as the exchange protein Epac. PKA signalling mediates diverse neuroprotective outcomes, such as phosphorylating CREB, promoting the transcription of BDNF and other pro-survival genes; it also regulates ion channels and neurotransmitter release, contributing to neural circuit stability, and it facilitates synaptic plasticity by phosphorylating AMPA receptors (as noted above) [40,42,87]. In glial cells, the cAMP/PKA pathway inhibits NF-κB activity, promoting anti-inflammatory effects. Many actions of GLP-1 in the brain (i.e., improved memory, reduced inflammation) are abolished if PKA is blocked, underscoring this pathway’s importance [88,89].

**AMP-Activated Protein Kinase (AMPK)**: AMPK functions as a cellular energy sensor, activated by increases in the AMP/ATP ratio. GLP-1R agonists can stimulate AMPK activity in neurons and glial cells, via upstream kinases or through CaMKKβ activation, which is triggered by calcium influx [90]. AMPK activation promotes autophagy and mitochondrial biogenesis. In PD models, GLP-1-induced AMPK activation was linked to enhanced mitochondrial gene expression and autophagic clearance of α-synuclein [91]. By activating AMPK, GLP-1RAs improve neuronal energy homeostasis and initiate cleaning of cellular debris, thereby protecting against metabolic stress.

These pathways do not operate in isolation but engage in extensive crosstalk; for instance, cAMP/PKA can feed into MAPK/ERK, and PI3K/Akt can interact with mTOR/AMPK signalling. The overall outcome of GLP-1R activation is an integrated coordination of pro-survival and restorative signals within neurons. This signalling profile, characterized by combining metabolic benefits with anti-apoptotic and anti-inflammatory signals, sets GLP-1RAs apart as multi-targeted therapeutic candidates for PD [41]. Importantly, these pathways are also active in peripheral tissues (e.g., Akt is involved in insulin signalling), which explains why drugs like exenatide can simultaneously improve systemic metabolism and brain cell health. Targeting such pathways has broad appeal and potential for modifying neurodegeneration [92]. Together, these signalling pathways demonstrate that GLP-1 receptor activation produces a convergent neuroprotective program in the brain in which metabolic restoration, trophic support, synaptic stabilisation, mitochondrial enhancement, and inflammatory suppression operate in parallel to counteract PD-related degeneration. GLP-1R-mediated stimulation of PI3K/Akt promotes cell survival through inhibition of pro-apoptotic proteins, upregulation of Bcl-2, stabilisation of mitochondrial membranes, and activation of PGC-1α-dependent mitochondrial biogenesis, effects first demonstrated in neuronal cultures by Li et al. and subsequently confirmed in multiple toxin-based PD models [9,41,57,79,86]. In tandem, engagement of the MAPK/ERK pathway enhances CREB phosphorylation and the transcription of neurotrophic factors such as BDNF and GDNF, supporting synaptogenesis, neurite outgrowth, and resilience against α-synuclein and oxidative injury [13,83,84]. The canonical cAMP/PKA axis further strengthens these responses by inducing CREB-dependent survival genes, regulating AMPA receptor trafficking, preserving dopaminergic firing patterns, and exerting anti-inflammatory effects through inhibition of NF-κB signalling in glial cells [40,42,87]. Concurrent activation of AMPK integrates metabolic stress responses by enhancing mitophagy, improving mitochondrial dynamics, and promoting clearance of damaged organelles and α-synuclein aggregates, thereby restoring energetic homeostasis in vulnerable dopaminergic neurons [9,48,91]. Crosstalk among these pathways amplifies their individual benefits, with PKA and ERK jointly sustaining CREB activity, Akt counteracting GSK-3β-mediated degeneration, and AMPK modulating mTOR activity to fine-tune autophagic flux. The combined consequence of these coordinated biochemical cascades is a robust, multimodal neuroprotective profile that addresses the interconnected metabolic, inflammatory, proteostatic, and mitochondrial disturbances central to Parkinson’s disease pathogenesis.

## 4. Key GLP-1 Agonists and Clinical Trials in PD

Several GLP-1R agonists have been tested for their potential to treat PD. Below are the key agents and highlights from preclinical and clinical studies available in the literature; Table 1 summarizes the key results of these studies.

### 4.1. Exenatide (Exendin-4)

Exenatide is the synthetic form of exendin-4 derived from Gila monster venom and was the first GLP-1RA tested in PD patients [77]. In rodent PD models (MPTP, 6-OHDA), exenatide demonstrated neuroprotective effects, preserving dopaminergic neurons and improving motor behaviour [98]. A landmark open-label trial in PD patients with moderate disease found that 12 months of exenatide (Byetta^®^, twice daily) led to significant improvements in motor and cognitive scores compared to controls, with benefits persisting 3 months post-treatment, suggesting potential disease-modifying effects [99]. A subsequent Phase II randomized controlled trial involving 60 patients, using once-weekly exenatide (Bydureon^®^) for 48 weeks, confirmed a relative improvement in off-medication motor scores (MDS-UPDRS part III) in the exenatide group, maintained even 12 weeks after cessation [5,93]. However, a Phase III trial conducted in 2020–2022, with 194 participants subjected to 2 years of treatment (2 mg of exenatide, once weekly), showed that both the placebo and exenatide groups presented worsening MDS-UPDRS part 3 scores at all timepoints [94]. The results of this last trial tempered early optimism, suggesting that exenatide’s benefits might be modest or limited to certain subgroups. Nonetheless, exenatide remains the prototypical GLP-1RA in PD research, demonstrating feasibility and some clinical signals of therapeutic effect in animal models. More studies, with larger samples and robust methodology, are required to properly elucidate its therapeutic potential in PD patients.

### 4.2. Liraglutide

Liraglutide is a long-acting GLP-1RA (once-daily human GLP-1 analog). It has strong preclinical support, showing protection of dopaminergic neurons and reduced neuroinflammation in MPTP and rotenone rodent models, with evidence of improved motor function and reduced α-synuclein aggregation [36,38]. Liraglutide has also readily improved cognitive function in animals [12,100]. Clinically, a Phase II trial in PD patients has been conducted and registered in clinicaltrials.gov (ID: NCT02953665); however, no official publication has been found and only a pre-print with the results is available (https://papers.ssrn.com/sol3/papers.cfm?abstract_id=4212371, accessed on 6 December 2025).

The study utilized three specific primary endpoints to assess motor, non-motor, and cognitive function changes from baseline at 28 and 54 weeks. Most of the outcomes presented did not significantly differ between the liraglutide and placebo groups. There was no therapeutic advantage on either motor or cognitive outcomes. Although the liraglutide group showed a modest 2.6-point improvement in the MDS-UPDRS part III motor scores, this was offset by a larger 5.0-point improvement in the other group, highlighting a more likely pronounced placebo response than any true drug effect. Similarly, MDRS-2 scores were no different between the study groups.

In the same way, many secondary and exploratory measures also failed to show a more visible advantage for liraglutide. Baseline metabolic markers did not predict treatment response, indicating that although liraglutide produced important reductions in BMI and HbA1c, these metabolic improvements did not translate into observable clinical benefits. Differently, there were a few positive findings, like a 6.6-point improvement of NMSS scores in the liraglutide group and a 6.5-point decrease in the placebo group. Despite the lack of clinical studies, liraglutide’s ability to improve both metabolic and neurologic parameters makes it an intriguing candidate [5,38]. Further larger trials are needed to confirm its efficacy.

### 4.3. Lixisenatide

Lixisenatide is a shorter-acting GLP-1RA, administered as a once-daily injection. Preclinical studies have demonstrated its ability to cross the BBB and exert neuroprotective effects [101]. In the Phase II randomized, double-blind “LIXIPARK” trial, 156 patients with early PD (≤3 years from diagnosis) received lixisenatide or placebo for 12 months [83]. Motor progression was measured by the change in the MDS-UPDRS Part III scores, which at baseline were approximately 15 in both groups. The lixisenatide group’s motor symptoms essentially stabilized, whereas the placebo group’s motor scores worsened; scores had changed by −0.04 points in the Lixisenatide group and +3.04 points in the placebo group, indicating imminent progression [83]. Even after a 2-month washout, patients previously on lixisenatide had better motor function than those who never received it. These results suggest lixisenatide might slow clinical decline in early-stage PD [95]. Lixisenatide also appeared to preserve brain cells, as inferred from stable dopamine imaging in exploratory endpoints. Notably, GI side effects were common, such as nausea in 46% of patients; however, this trial provides a proof of concept that a GLP-1RA can slow PD motor progression and that larger Phase III trials will be needed to verify disease modification and long-term results.

### 4.4. Semaglutide

Semaglutide is a potent, long-acting GLP-1RA available as a weekly injection (Ozempic^®^) or as an oral formulation. Its higher molecular weight gives it a longer half-life but somewhat lower BBB penetration than exenatide; despite this, semaglutide has shown efficacy in animal models. In a chronic MPTP mouse model, semaglutide not only improved motor performance but also reduced nigral α-synuclein accumulation, lowered oxidative stress markers, and increased dopaminergic neuron survival. In many of these measures, semaglutide performed as well as or better than liraglutide [44]. These findings position semaglutide as a very promising agent for PD. Additional studies report modulation of apoptotic and autophagy-related pathways; semaglutide effectively restored Bcl-2 levels, which were previously reduced by MTPT exposure, and decreased Bax levels [23]. While both drugs, semaglutide and liraglutide, partially lowered the Bax/Bcl2 ratio by increasing Bcl2 and reducing Bax expression significantly (*p* < 0.001), semaglutide demonstrated to have superior anti-apoptotic efficacy; it could also rescue autophagy impairments seen through a higher expression of beclin1, Atg7, LC3, and P62 in the substantia nigra [23]. Clinically, a Phase II trial (NCT03659682) is enrolling patients to receive weekly semaglutide for up to 2 years, with primary motor outcomes evaluated and possibly exploratory neuroimaging biomarkers [97]. As of 2025, no published clinical results are available yet for semaglutide in PD. However, given its strong glycaemic control profile and neuroprotective preclinical data, semaglutide is anticipated to be a relevant player in upcoming PD trials. One advantage is its convenient dosing schedule (once-weekly injection) and the availability of an oral formulation, which could improve long-term adherence in the PD population.

### 4.5. Other Agents

The dual agonist DA5-CH and NLY01, a pegylated formulation of exenatide, have each shown superior neuroprotective effects in PD models.

Nevertheless, the current development status of DA5-CH remains at the preclinical stage. It has been extensively studied in various animal models, including the MPTP mouse model and the 6-OHDA lesion rat model. Both investigations aimed to evaluate its effects on motor impairments, inflammation, dopamine synthesis, and alpha-synuclein levels [59,102].

In contrast, a Phase II clinical trial determined that by week 36, NLY01 showed no difference from placebo in MDS-UPDRS parts II and III scores: −0.39 (95% CI −2.96 to 2.18, *p* = 0.77) for 2.5 mg and 0.36 (−2.28 to 3.00, *p* = 0.79) for 5.0 mg [29].

In a comparative study, one-way ANOVA analysis revealed that DA5-CH managed to recover 79.61% of TH-positive cells, which had been significantly reduced to 39.65% in the MPTP group. In contrast, NLY01 only rescued 48.62% of the cells [59].

Additionally, DA5-CH was shown to inhibit the TLR4/TNF-α-mediated inflammatory pathway more effectively than NLY01. While MPTP elevated the expression of TLR4, NF-kB, and TNF-α, DA5-CH downregulated all three markers, whereas NLY01 only reduced NF-kB and TNF-α, not TLR. In general terms, this demonstrated that the novel dual GLP-1/GIP receptor agonist DA5-CH had a much better performance than NLY01 [59]. Additionally, another dual incretin receptor agonist, tirzepatide, is routinely included in preclinical pharmacokinetic studies to determine its promise as a therapeutic for neurodegenerative diseases. It has been associated with a substantial decrease in the BAX/Bxl-2 ratio, supporting neuronal survival; likewise, histological analysis showed that it protected tyrosine hydroxylase-positive neurons, maintaining the integrity of the nigrostriatal dopaminergic pathway [103]. The treatment not only led to significant behavioral improvements but also attenuated neuroinflammation in a rotenone-induced rat model of Parkinson’s disease [104]. Overall, the studies suggest that tirzepatide has disease-modifying potential, although no clinical trials yet confirm these effects. Even so, the study on brain uptake pharmacokinetics showed that tirzepatide has poor BBB penetration, especially in the first hour, implying that it is not efficiently transported into the brain. This limitation may be considered a challenge to its effectiveness in treating central diseases like PD [105].

Other potential agents are dipeptidyl peptidase-4 (DPP-4) inhibitors; DPP-4 is an enzyme that primarily inactivates incretin hormones like GLP-1 and GIP, which are involved in regulating insulin secretion and blood glucose levels. DPP-4 inhibitors boost endogenous GLP-1 and could be another potential treatment avenue, with supporting epidemiological evidence of reducing PD risk [4]. A longitudinal population study included 329 patients diagnosed with PD with a median follow-up of 3.33 years; the incidence of PD was 5 per 10,000 person-years in 36,897 patients using DPP4 inhibitors and 4 per 10,000 person-years in 10,684 using GLP-1 agonists. Interestingly, they reported an important inverse association between use of DPP4 inhibitors and GLP-1 agonists and the onset of PD, with an adjusted incidence rate ratio of 0.64 (95% CI, 0.43–0.88; *p* < 0.01) for DPP-4 inhibitors [4]. Another preclinical study of DPP4 inhibitors, also known as gliptins (sitagliptin and PF-00734,200), that used the 6-OHDA rat model of PD revealed several beneficial findings, such as dopaminergic cell preservation and DA level protection [106]. Delayed oral treatment with a higher dose of sitagliptin increased dopamine levels in both the lesioned striatum and SNc; similarly, it also augmented neurogenesis and increased BrdU labelling (a marker of cell proliferation/DNA synthesis) in the lesioned striatum [106]. This suggests that they may enhance the migration of NPCs (neuronal progenitor cells) to the lesioned striatum. Despite the remarkable benefits found, the study also highlighted some limitations. For a start, it has been proven that sitagliptin has a low brain access rate (brain/plasma total ratio of 0.07), likely due to it being a substrate for the P-glycoprotein efflux transporter at the cerebral microvasculature. In contrast, PF-00734,200 showed a higher concentration in the rat brain (brain/plasma total ratio of 0.62) [106]. However, there is an efficacy disparity since sitagliptin treatment was more potent than PF-00734,200 in rescuing dopaminergic neurons and protecting against dopamine loss [106]. Another constraint detected is that although gliptins increased BrdU labelling, the newly proliferating cells did not express TH (a marker for dopaminergic neurons), a fact that requires further investigation. Clinical studies need to be performed to properly ascertain their treatment potential.

### 4.6. Relevant Dosage and Pharmacokinetic Considerations

The dosage and evaluation of the neuroprotective effects of GLP-1RAs in PD are based on extrapolation of the efficacy observed in preclinical models and on standardized dosage guidelines for type 2 diabetes mellitus (T2DM) [107,108]. Exenatide, using a subcutaneous dose of 2 mg, identical to the licensed dose for T2DM, has demonstrated its ability to cross the BBB and be detectable in cerebrospinal fluid at concentrations similar to those associated with beneficial outcomes in preclinical models [5,93,94,99]. In a randomized, double-blind, placebo-controlled trial, weekly exenatide showed a significant advantage in the primary outcome: the adjusted difference in the motor subscale (part III) of the Movement Disorders Society Unified Parkinson’s Disease Rating Scale (MDS-UPDRS). Furthermore, a previous 12-month study also reported an improvement in the MDS-UPDRS of 2.7 points in patients treated with exenatide, compared with a decline of 2.2 points in controls. These findings demonstrate an initial advantage by not requiring a modification of the licensed dose to achieve central access and motor efficacy in PD [5,93,94,99].

The interest in exenatide lies precisely in its favorable pharmacokinetics, as a non-acylated molecule, it has a higher and more efficient rate of penetration of the BBB than its analogues such as semaglutide, evaluated in trials using its licensed doses of 0.5–2 mg weekly (subcutaneous, Ozempic) or 3–14 mg daily (oral, Rybelsus) or liraglutide; both are acylated analogues and their structure gives them a high affinity for albumin and strong plasma protein binding, which significantly restricts their distribution and effective concentration in the central nervous system [109]. Nevertheless, in the randomized, double-blind, placebo-controlled trial of Liraglutide, administered at doses of 1.2 mg or 1.8 mg per day, the primary endpoint was measured by the adjusted difference in the MDS-UPDRS motor scale in the OFF-state, despite the limitations of its central penetration [96,110].

The advantage of exenatide in terms of access to the central nervous system highlights its relevance as a GLP-1RA initially more suitable for PD research. However, the limitations observed with its acylated analogues reinforce the importance of overcoming the pharmacokinetic obstacle for the future of this therapeutic line. For the activation of GLP-1 receptors translatable into the desired effects (improve mitochondrial function, reduce neuroinflammation, and neuronal survival), dosage optimization or molecular modifications are required to allow more efficient passage of the BBB. Thereby, achieving higher concentrations and a direct neuronal effect with the aim of converting neuroprotective promise into tangible clinical benefit in PD.

A consolidated overview of the principal preclinical studies evaluating GLP-1 receptor agonists in Parkinson’s disease models, including mechanistic findings, experimental systems, dose considerations, and translational relevance, is provided in Table 2.

## 5. Conclusions and Future Directions

GLP-1 receptor agonists represent a novel therapeutic strategy in PD by targeting the complex interplay of metabolic, inflammatory, and degenerative mechanisms underlying it. Extensive preclinical evidence indicates that GLP-1 signalling can counteract key pathogenic processes, from neuroinflammation and oxidative stress to protein aggregation, and thereby confer neuroprotection to dopaminergic neurons. Clinical trials to date have delivered mixed but encouraging results; while not yet definitive, some studies suggest that GLP-1RAs may slow the clinical decline in PD or improve certain symptoms beyond what standard therapies achieve. However, the recent negative Phase III trial of exenatide reminds the field that translating neuroprotective effects to tangible patient benefits is challenging [5,67,94]. Likewise, clinical trials evaluating the efficacy of NLY01 in early, untreated Parkinson’s patients did not yield favourable results [29]. These highlight the need for optimizing patient selection (e.g., earlier disease stage might provide more impact, as hinted by the positive early-PD lixisenatide trial [95]), and optimizing the pharmacokinetics or brain penetration of these drugs is perhaps a potential avenue that should be explored.

Despite the solid mechanistic basis and consistent preclinical evidence, several rate-limiting issues limit the clinical efficacy of GLP-1RAs in PD. The main limitation, as previously mentioned, lies in the differential pharmacokinetics of GLP-1RA analogues, particularly in their ability to penetrate the BBB. Smaller, non-acylated analogues such as Exendin-4 show more efficient penetration in contrast with acylated molecules with higher molecular weight, such as liraglutide and semaglutide, which have shown slow and limited transport due to their high affinity for albumin, which potentially reduces their direct action on dopaminergic neurons [7,20,66,101]. This raises the question of whether current analogues reach the necessary therapeutic concentrations in the brain. A second limitation is the biological heterogeneity of PD, where not all patients share the same degree of neuroinflammation, symptom progression, mitochondrial dysfunction, or α-synuclein accrual. Therefore, the response to GLP-1RAs likely varies between subgroups, and this biological variability calls attention to the need for precision medicine, dose adaptation, and the identification of subgroups that may benefit from its metabolic modulation [11,93]. In addition, it is necessary to consider the timing of intervention; most clinical trials included patients in moderate or advanced stages of PD, which could explain such negative results as those of the exenatide trial in phase III [94]. At these stages, there is already an irreversible degree of dopaminergic neuronal loss, minimizing the potential for neuroprotective therapy. The LIXIPARK trial with lixisenatide, focusing on patients in the early stages (≤3 years), showed a delay in clinical deterioration, reinforcing the hypothesis that intervention must occur early to achieve disease modification [95]. Furthermore, guideline doses used in TDM2 may not be sufficient to achieve the brain concentrations required to maximize the neuroprotective effect in PD, suggesting that pharmacokinetic adjustments or structural optimization would be necessary to maximize the neuroprotective effect, either through higher tolerable doses or preferably through structural optimization of analogues to improve brain access, as is being explored with dual GLP-1/GIP agonists.

It is important to note that although GLP-1RAs possess a well-characterized safety profile in T2DM and obesity, some adverse effects gain greater relevance in PD. The most common reactions include nausea, vomiting, weight loss, and decreased appetite, reported in 30–50% of patients in trials with exenatide or lixisenatide [4,11]. Similarly, it can exacerbate pre-existing conditions such as dysphagia, gastroparesis, or low body mass. Other reported events include diarrhea, constipation, and headache. No significant adverse neuropsychiatric effects have been observed, but some trials have reported fatigue and reduced appetite as causes for discontinuation. Whereas GLP-1RAs do not appear to worsen motor symptoms, it is important to monitor orthostatic hypotension, as weight loss and reduced intake can aggravate it in PD. Overall, tolerability is acceptable; a 2022 TDM2 retrospective cohort demonstrated that gastrointestinal side effects were the most frequent adverse events and occurred in higher proportions in patients receiving exenatide during the entire follow-up period. A total of 23.7% patients discontinued treatment, mainly due to limited effectiveness in lowering HbA1c or gastrointestinal side effects [5,29,95,111].

The need to optimize clinical efficacy extends to trial methodology, making the selection of endpoints critical for detecting a true neuroprotective effect, while sensitivity to motor symptom changes is insufficient to capture the underlying biological progression. Therefore, a comprehensive approach incorporating imaging biomarkers such as DaT-SPECT or PET-FDOPA to quantify nigrostriatal dopaminergic function, PET of microglia to measure neuroinflammation, and volumetric MRI to detect striatal or nigral atrophy is strongly recommended [112]. At the level of biological biomarkers, monitoring phosphorylated a-synuclein in plasma or CSF and neurofilament light is essential as they are indicators of neurodegeneration, complemented by inflammatory markers [69,113]. In addition, the evaluation of robust functional endpoints, such as activities of daily living (MDS-UPDRS II), is crucial. Moreover, given the nature of GLP-1RAs, specific metabolic endpoints should be included, such as measures of insulin resistance, weight, and body composition, which may help to profile responders. This combined approach of symptomatic, functional, and biological endpoints is essential to determine whether GLP-1RAs exert disease-modifying effects rather than merely symptomatic ones [30,72,114].

Therefore, the identification and stratification of patients who could benefit most from GLP-1RAs is imperative for the success of future trials. Epidemiological and mechanistic evidence suggests that individuals with DM2, prediabetes, or insulin resistance are at increased risk of PD progression and may therefore respond more favorably to GLP-1RAs, given the dual metabolic action of these drugs [4,8]. Consequently, patients with elevated HOMA-IR, metabolic syndrome, or a high BMI may constitute a subgroup that is particularly likely to benefit. Above all, candidate selection must be cautious; patients with low weight, severe dysphagia, gastroparesis, or gastrointestinal motility disorders may be poor candidates due to the increased risk of nausea, weight loss, and intolerance, which could lead to high discontinuation rates. Furthermore, clinical experience indicates that patients with advanced PD, in whom dopaminergic loss is extensive, are likely to derive limited benefit, as observed in the failed phase III trial with exenatide. Evidently, to maximize clinical efficacy and reduce discontinuations due to tolerability, candidate selection could be supported by a comprehensive assessment including metabolic profiles, inflammatory markers, clinical phenotypes, and, eventually, validated biomarkers. This stratified approach is crucial to align the therapeutic potency of GLP-1RAs with the biological heterogeneity of PD.

Future research is increasingly directed toward next-generation incretin mimetics, such as dual GLP-1/GIP agonists and blood–brain-barrier-optimized analogues, with the aim of enhancing central efficacy [59]. Combining GLP-1RAs with complementary neuroprotective approaches (e.g., anti-synuclein immunotherapy or exercise) may provide synergistic benefits. Moreover, deciphering patient subgroups, for instance, PD patients with diabetes or insulin resistance who might respond differently to GLP-1 therapy [80], will likely be key for implementing personalized treatment strategies. Evidence from animal models suggests that addressing such comorbidities with GLP-1 drugs can lead to notable neuroprotective effects [115].

As scientists continue to unravel GLP-1’s actions in the brain, there is optimism that this line of therapy, already safe and in clinical use for diabetes, can be repurposed or refined to alter the course of PD [28]. Large, well-powered trials are in progress to definitively assess disease modification by GLP-1RAs. Even if the degree of benefit is moderate, GLP-1RAs could become part of eventual multimodal therapeutic strategies aimed at the multiple facets of PD pathology (metabolic dysfunction, inflammation, etc.), meriting their place in the quest for neuroprotective Parkinson’s therapies.

## Figures and Tables

**Table 1:** Summary of clinical trials evaluating GLP-1R agonists in PD.

Agent	Phase	Duration	n	Outcome Measure	Key Result	Reference
Exenatide	II	48 weeks	60	MDS-UPDRS III	Improved 3.5 points vs. placebo	Athauda et al., 2017 [93]
Exenatide	III	2 years	194	MDS-UPDRS III	No difference	Vijiaratnam et al., 2025 [94]
Lixisenatide	II	12 months	156	MDS-UPDRS III	Stabilised symptoms vs. worsening	Meissner et al., 2024 [95]
Liraglutide	II	52 weeks	70	NMSS	Improved non-motor symptoms	SSRN preprint, 2024 [96]
Semaglutide	II	Ongoing	—	—	Pending	NCT03659682 [97]

**Table 2:** GLP-1 receptor agonists in preclinical models of Parkinson’s disease and their translational relevance.

Drug/Class	Preclinical PD Model	Main Findings	Dose/Regimen (Qualitative)	Translational Relevance and Major Caveats	References
**Exendin-4 (Exenatide)**	6-OHDA rat; MPTP mouse; rotenone cell models	Preserves TH-positive neurons; restores striatal dopamine; suppresses microglial activation and inflammatory cytokines; enhances mitophagy; and preserves mitochondrial membrane potential under rotenone stress.	Systemic dosing often higher (mg/kg) than human antidiabetic exposures; typically administered close to toxin exposure.	Robust neuroprotection in toxin models, although these models have limited construct validity; dosing often supra-therapeutic relative to humans; Phase III clinical trial showed no benefit despite strong preclinical results.	[9,11,36,42,43,56,61]
**Liraglutide**	MPTP mouse; rotenone models	Reduces α-synuclein aggregation; improves mitochondrial complex I function; decreases inflammatory markers; and improves motor outcomes.	Once-daily injections at or slightly above T2DM dose ranges; limited BBB penetration due to acylation and albumin binding.	Mechanistic benefits strong but human results (preprint only) show no clear clinical advantage; limited CNS access may restrict translation.	[9,12,36,38,100]
**Semaglutide**	Chronic MPTP mouse	Improves motor behaviour; reduces nigral α-synuclein; enhances dopaminergic neuron survival; boosts autophagy (beclin-1, Atg7, LC3) and anti-apoptotic signalling (↑ Bcl-2, ↓ Bax).	Weekly dosing at high systemic exposures compared with human use.	Very promising mechanistic profile but limited BBB penetration and no human PD results yet; translational predictions remain preliminary.	[23,44,48]
**PT320 (Sustained-release exendin-4)**	MitoPark mouse; 6-OHDA and MPTP models	Preserves mitochondrial ultrastructure (Opa1/Fis1); maintains striatal dopamine release and reuptake; delays motor decline; reduces L-DOPA-induced dyskinesia (lower ALO, limb and orolingual AIM scores).	Extended-release exposure; often initiated early in disease course, not reflective of typical clinical PD.	Benefits demonstrated in a genetic PD model with higher construct validity; however, exposures are supra-physiological and human PK is still uncertain.	[45,46]
**Lixisenatide**	MPTP mouse; inflammation-associated models	Crosses BBB; improves synaptic markers and cognitive-relevant pathways (NTRK2, mTOR); reduces neuroinflammation.	Daily systemic dosing at similar or modestly higher exposures than T2DM regimens.	Clinical Phase II LIXIPARK trial demonstrated slowed motor progression in early PD, consistent with preclinical findings; GI effects common.	[10,83,95,101]
**NLY01 (Pegylated exendin-4)**	α-synuclein PFF models; LPS-induced neuroinflammation	Blocks microglia-driven A1 astrocyte conversion; protects dopaminergic neurons; improves behavioural readouts.	Pegylated formulation with prolonged half-life and high steady exposure.	Strong glia-targeted mechanism but Phase II clinical trial showed no benefit.	[19,28,29,30]

## Data Availability

No new data were created or analyzed in this study. Data sharing is not applicable to this article.
